# Relationship Between Regional Atherosclerosis and Adjacent Spinal Cord Histology

**DOI:** 10.7759/cureus.329

**Published:** 2015-09-21

**Authors:** R. Shane Tubbs, Matthew C Blouir, Rajani Singh, Nirusha Lachman, Anthony V D'Antoni, Marios Loukas, Eyas Hattab, Rod J Oskouian

**Affiliations:** 1 Neurosurgery, Seattle Science Foundation; 2 Seattle Science Foundation; 3 Department of Anatomy, AIIMS, Virbhadra Marg, Pashulok, Rishikesh; 4 Department of Anatomy, Mayo Clinic; 5 Department of Pathobiology, The Sophie Davis School of Biomedical Education; 6 Department of Anatomy, St. George's University; 7 Department of Pathology and Laboratory Medicine, Indiana University; 8 Department of Neurosurgery, Swedish Neuroscience Institute

**Keywords:** anatomy, spinal cord, neurons, blood supply, atherosclerosis, arteries, plaque disease

## Abstract

Introduction: Scant data are available regarding ischemic insult to the spinal cord and the responsible blood supply. Therefore, we aimed to investigate a correlation between atherosclerosis of adjacent vessels and spinal cord ischemia.

Materials and Methods: In 20 unembalmed adult cadavers, samples of the vertebral arteries and aorta were removed and the degree of atherosclerosis with subsequent luminal occlusion was histologically analyzed. Next, adjacent segments of the spinal cord were harvested and submitted for immunohistological analysis of both neural and glial elements and blood supply.

Results: We identified proximal atherosclerosis in the majority of cadavers but with varying degrees of luminal occlusion. The greatest degree of luminal occlusion was found in the descending abdominal aorta. No specimen was found to have atherosclerosis of the anterior or posterior spinal or radicular arteries. No spinal cord histology showed signs of ischemia, even in specimens with a significant large parent vessel (vertebral artery and aorta) occlusion due to atherosclerosis. Neuropathology of these adjacent cord segments revealed no signs of ischemia or demyelination.

Conclusions: Spinal cord ischemia is often misdiagnosed and can cause significant neurological compromise. However, based on our study, the degree of atherosclerosis of the adjacent parent vessel supply does not appear to be  a predictor of neuronal and glial tissue damage of the adjacent spinal cord.

## Introduction

Heart disease caused by atherosclerosis is the primary cause of death for both men and women in the United States [1-2]. In 2004, the estimated total number of Americans suffering from atherosclerotic-related heart disease was approximately 80 million, with roughly half of that patient population being over 65 years old [1]. In 2004, the mortality rate due to atherosclerosis was one in every 2.75 deaths, or 36.3% of all deaths in the U.S. [1]. The American Heart Association estimated that over $400 billion from direct and indirect costs can be attributed to atherosclerotic disease [1]. Furthermore, by the year 2040, statistical estimates predict that 20% of the U.S. population will be over the age of 65 [3]. 

Although coronary artery and peripheral arterial disease due to atherosclerosis have been well studied, there are few data on the ischemic pathology of the spinal cord secondary to vascular occlusion. This is despite the fact that areas of the spinal cord that “soften” due to atherosclerosis were first described as early as the late 19th century [4-5]. Like atherosclerosis, myelopathies from focal necrosis, Wallerian degeneration, and neuronal atrophy increase in incidence and severity with age [4, 6]. These neural degradations can manifest as unexplained local and generalized paresthesia, paresis, muscular atrophy, pain, urinary incontinence, diminished proprioception and balance, hyperreflexia or diminished tendon reflexes, or any number of symptoms that are commonly attributed to “old age” [4, 6]. Such symptoms of neural deterioration may persist without being attributed to a specific neurological disease or motoneuron disorder [4, 6].  

From an anatomical standpoint, the current knowledge and understanding of atherosclerosis has provided support to the hypothesis that atheromatous plaque formation tends to occur more frequently near arterial bifurcations [7-9]. Due to disruptions in normal laminar blood flow at an arterial division, shear stress exerted against the tunica intima is said to cause endothelial injury and inflammatory response [7-9]. Due to the numerous bifurcations through which blood must pass to perfuse the spinal cord and the “looped and tortuous” nature of these vessels, this understanding supports the theory that degenerative changes of spinal neurons may be correlated with atherosclerotic changes in the vasculature that perfuse the spinal cord [10]. Despite this association, few studies have been conducted to evaluate the existence of such a correlation between these two variables. Most of the literature available comes from case studies and descriptions of the spinal cord blood supply that date back to the early and mid-20th century, in which only speculative conclusions and untested hypotheses were made [4-5, 10-18].  Of the limited amount of research that exists, most of it appears to provide support for the existence of a correlation; however, the majority of these data are greater than three decades old [4-6]. Furthermore, the preponderance of the existing literature has been conducted in sample populations from various European countries (Germany, France, England, Italy, and the Netherlands) and Japan [4-5, 10-17]. The paucity in the current literature necessitates further research utilizing more current specific histological techniques to help establish or refute a pathological relationship between systemic atherosclerosis and spinal myelopathy. The growing impact of atherosclerosis on the U.S. population as well as the lack of population-specific data warrants further investigation into whether a correlation exists between the level of systemic atherosclerosis and spinal cord ischemia in adult human cadavers from the U.S. population. 

## Materials and methods

Our paper involved the study of human tissue from cadavers, and therefore, informed patient consent was waived for this study. No identifying patient information was disclosed in this paper. 

Of the total number of cadavers utilized for this study (n=20), 12 were male, and eight were female. The mean age (range) at the time of death for the subjects was 75 (69 - 103) years. The causes of death ranged from chronic obstructive pulmonary disease to pancreatic cancer. No specimen had known pathology or past surgery of or near the spinal cord.

### Procedures

Once a cadaver was randomly selected (out of a total number of 50 cadavers) for dissection, seven arterial/cord specimens were harvested and preserved in formalin. These seven specimens consisted of samples taken from the left and right vertebral arteries, thoracic aorta, abdominal aorta, cervical spinal cord, thoracic spinal cord, and the conus medullaris. 

### Vertebral artery samples

The cadaver was placed in the supine position, and an incision was made from the mental protuberance to the jugular notch, which joined two other incisions made along the inferior border of each clavicle. All superficial tissues were reflected to allow for disarticulation and removal of the clavicles. Once the clavicles were removed, the proximal 1 cm portion of the right vertebral artery (V1 segment) was identified at its origin from the right subclavian artery and excised with dissecting scissors, labeled with a surgical marking pen, and placed in a 10% formalin solution. The same process was repeated for the harvesting of the left vertebral artery, which was labeled and fixed in solution accordingly. 

### Aortic samples

Next, with the body supine, a sternal incision was made, and the skin and overlying musculature reflected to allow for removal of the anterior thoracic wall. Once the aorta was exposed, a 1 cm long sample was taken 2 cm distal to the origin of the left subclavian artery. This sample was then labeled and fixed for evaluation.

Lastly, a midline incision was made from the epigastric region to the pubic symphysis, which allowed access to the abdominal cavity. After retracting and/or removing superficial peritoneal structures, the distal abdominal aorta was identified and mobilized. A 1 cm long sample of the abdominal aorta was taken just superior to the aortic bifurcation and then marked and fixed in solution.

### Spinal cord segments

For each cadaver, three spinal cord segments were collected for analysis from the cervical and thoracic regions and the conus medullaris. All segments were excised with their associated spinal nerve roots, radicular arteries, and spinal vasculature intact. No effort was made to specifically identify which radicular artery was the artery of Adamkiewicz. Once the vascular specimens mentioned above were harvested, the cadaver was rotated into the prone position, and an incision was made from the occiput to the sacrum. The overlying soft tissues were then reflected to allow for visualization of the posterior aspect of the vertebral column. A laminectomy was performed from C1 through L5 exposing the dura mater. A midline incision was then made along the full length of the dura mater, which was then reflected laterally. Three specimens of spinal cord, each measuring 1 cm in length, were then excised with their associated spinal nerve roots, radicular arteries, and spinal vasculature intact between C2 and C3 (labeled cervical segment), from the level of T6 (labeled thoracic segment), and from the conus medullaris (labeled conus medullaris segment). 

### Measurements/histology

The four arterial specimens from each cadaver were evaluated for the degree of atheromatous plaque formation and the data recorded so that the total percent occlusion of the arterial lumen was determined. The area of each arterial lumen was calculated and then the decrease in the area (occlusion) calculated. Using the equation for the area of a circle, a decrease in the area was calculated and expressed as a percentage. The spinal cord specimens were sliced into 5-micron sections. Standard and immunohistochemical stains that included hematoxylin and eosin, Luxol fast blue-Periodic acid Schiff, glial fibrillary acid protein, Verhoeff and Masson trichrome stains, were used to evaluate for focal necrosis, ischemic changes, and nuclear and white matter degeneration/neuronal cell loss.

## Results

**Figure 1:**
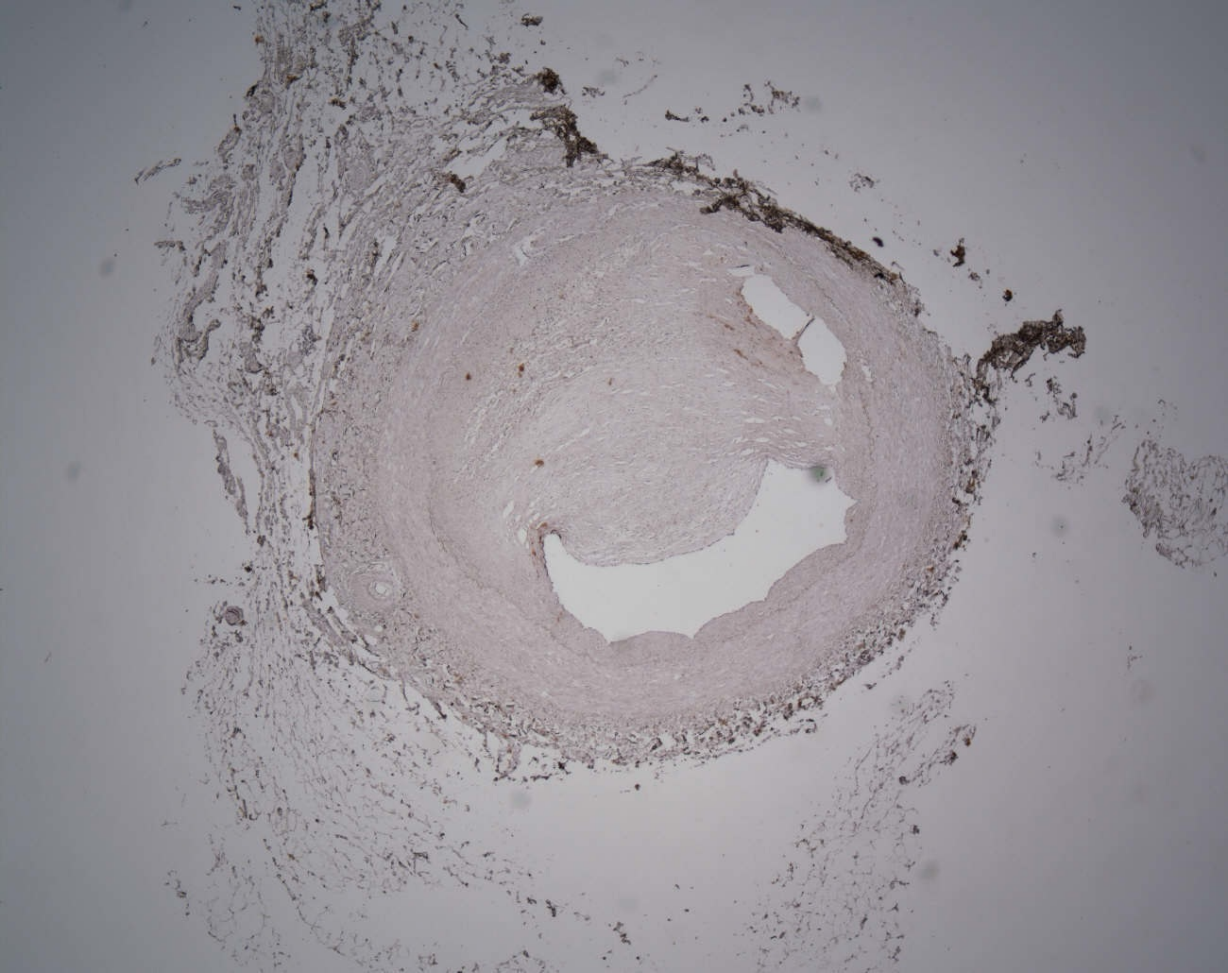
Histological specimen (axial section x 50) through left vertebral artery This image illustrates significant vascular stenosis due to plaque disease.

**Figure 2:**
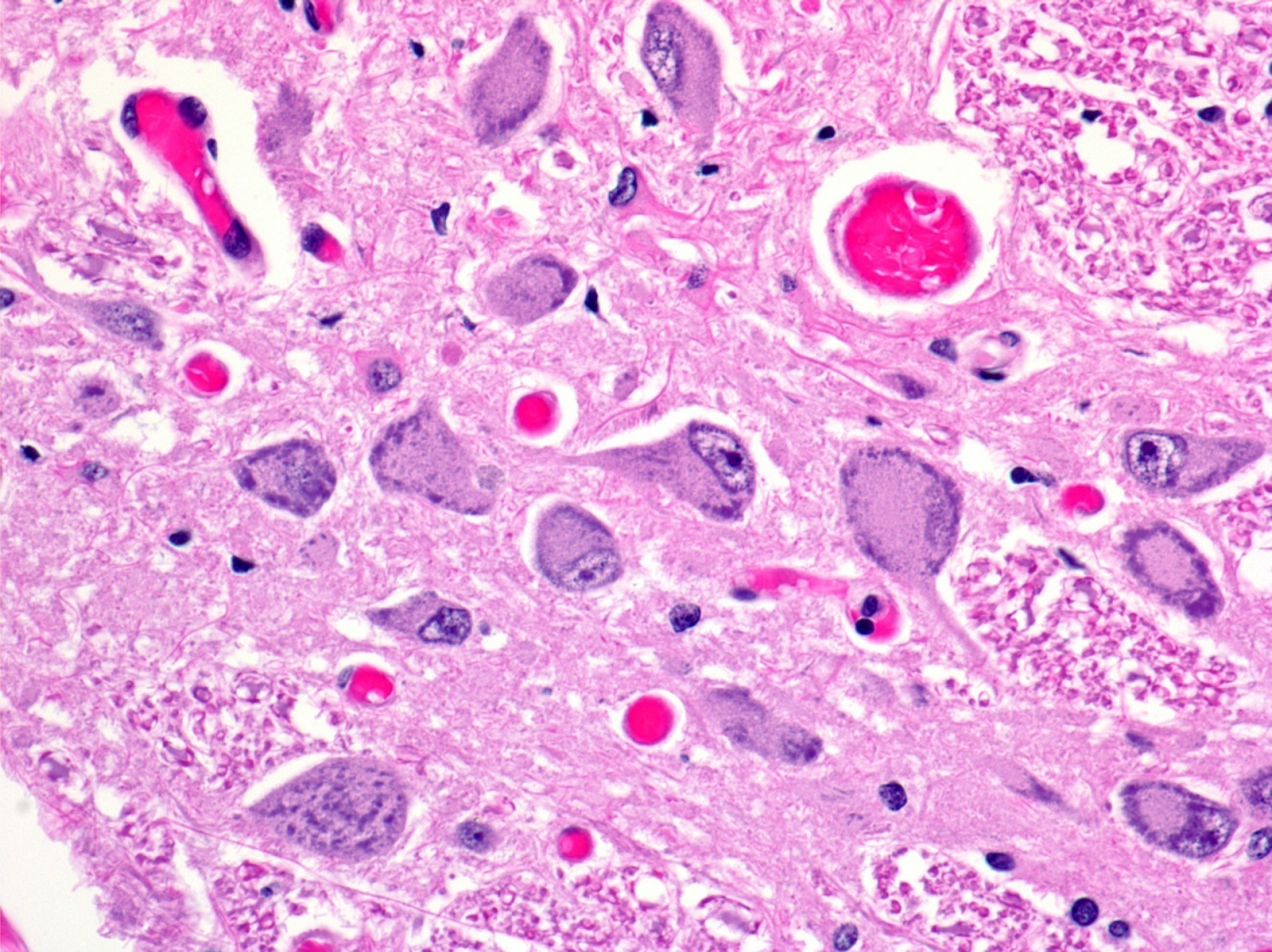
Adjacent spinal cord histologic section (axial section x 400) This section demonstrates normal neurons and counts from central gray matter of the spinal cord from specimen shown in Figure 1.

**Figure 3:**
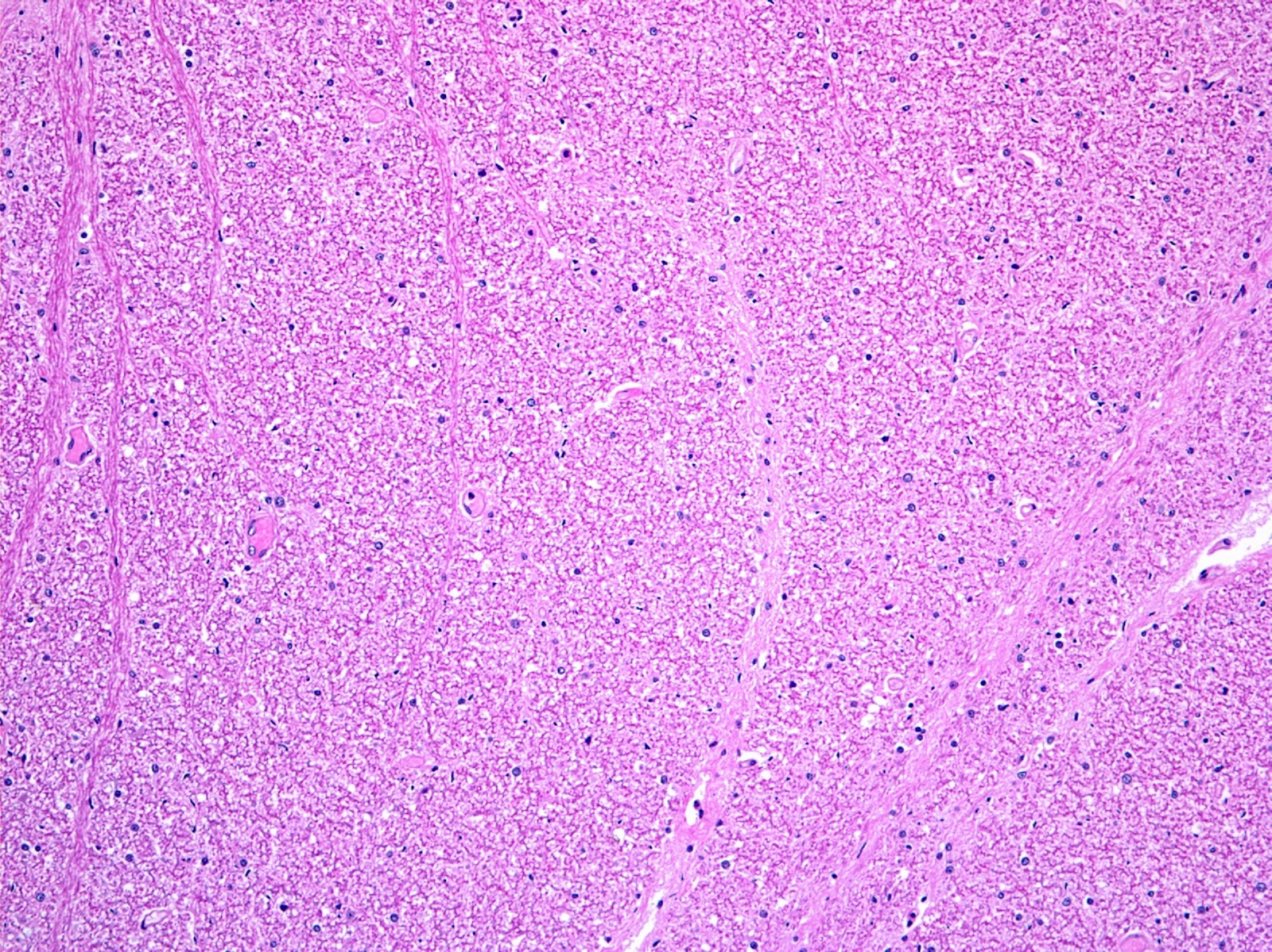
Adjacent spinal cord histologic section (axial section x 100) This section also from the specimen shown in Figure 1 demonstrates normal white matter.

**Figure 4:**
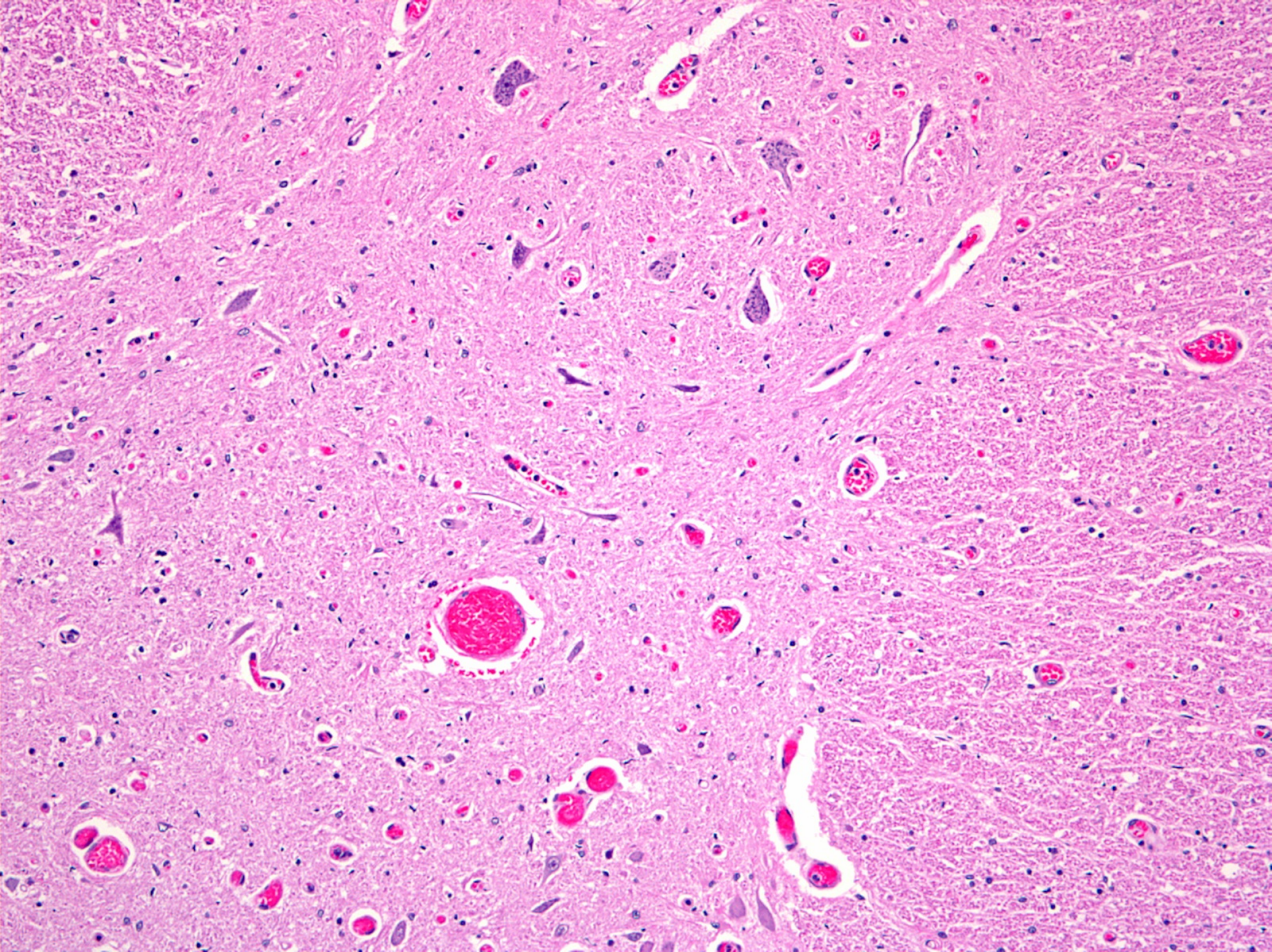
Adjacent spinal cord histologic section (axial section x 100) This image illustrates gray and white matter of the adjacent spinal cord and the rare reactive astocytosis seen in some specimens.

No cadaver had a known history of spinal cord, limb dysfunction, or neurologic diseases. Grossly, no obvious abnormalities were found in the harvested spinal cord segments. For thoracic and lumbar segments, rare reactive astrocytes were observed and a subtle increase in polyglucosan bodies (consistent with aging) was seen around some cord vessels and in an increasing frequency from the cervical to thoracic and lumbar cord segments. Such astrocytes were not seen in any of the cervical cord segments and without macrophages or axonal swelling. The degree of vessel occlusion was 0-80% (left vertebral artery), 0-55% (right vertebral artery), 5-45% (thoracic aorta), and 10-50% (abdominal aorta). In two lumbar specimens (one male and one female), a mild focal reactive gliosis was seen. There was no correlation between large degrees (30-80%) of atherosclerosis in adjacent arteries and these subtle increases in reactive astrocytes and polyglucosan bodies (Figures 1-4). Some rare neuronal vacuolization was observed. However, these were more consistent with age or artifact than ischemia. These areas of vacuolization were also not correlated to the degree of atherosclerosis of adjacent arteries. No signs of inflammation were seen in any of the specimens. One female specimen aged 95 years at death was found to have a mild loss of myelin in the cervical and thoracic posterior columns consistent with poor nutrition (e.g., a vitamin B12 deficiency). Spot neuronal counts for all levels analyzed were within normal limits compared to histologic controls.

## Discussion

### Spinal cord ischemia and infarction

**Figure 5:**
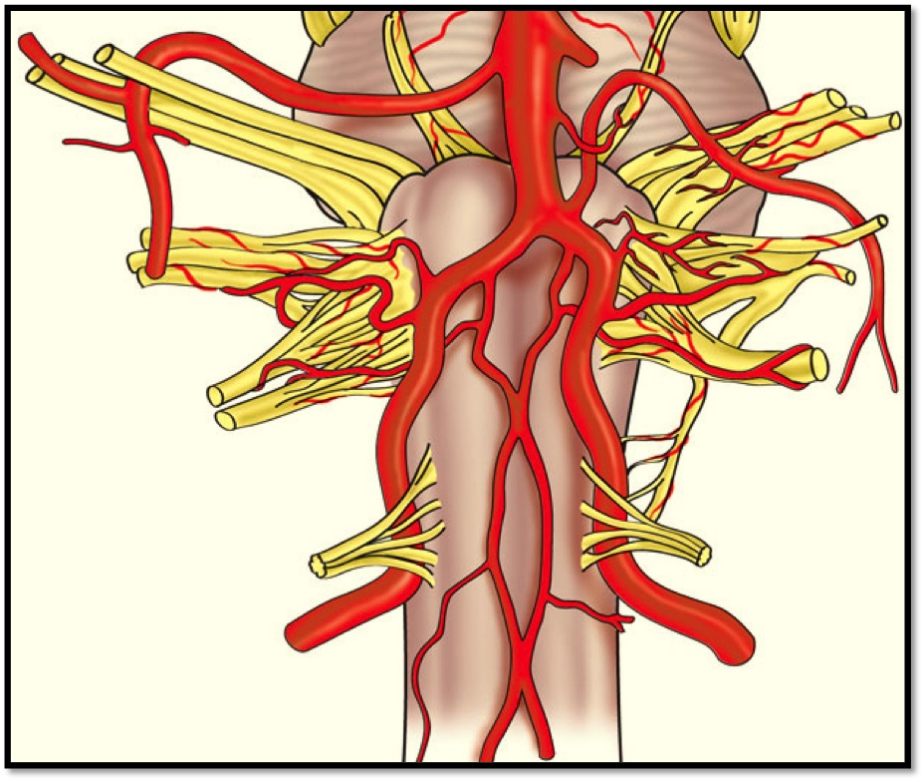
Anterior view of the proximal spinal cord and brain stem Schematic drawing of the blood supply of the upper anterior spinal cord from the anterior spinal artery shown here derived from the vertebral arteries.

**Figure 6:**
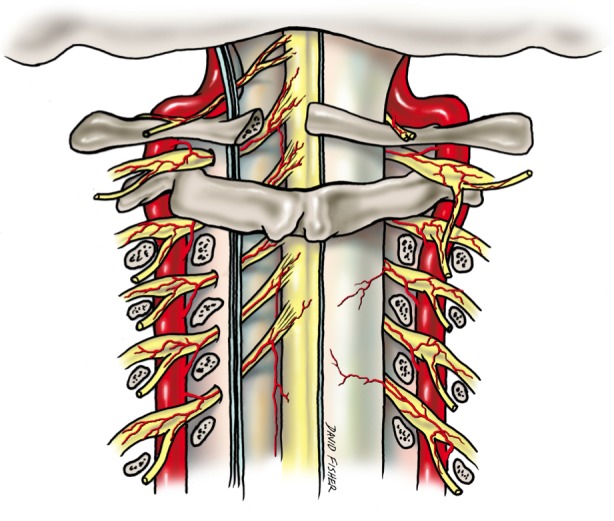
Posterior view of the proximal spinal cord Schematic drawing of the blood supply to the posterior aspect of the spinal cord via radicular branches of the adjacent vertebral arteries.

**Figure 7:**
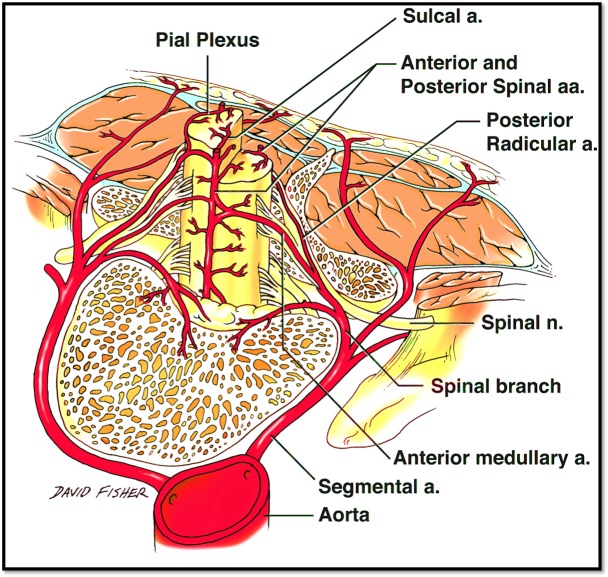
Anterosuperior view of the spinal cord Schematic drawing of the blood supply to the spinal cord from the adjacent aorta.

Ischemia and infarction of the spinal cord (the so-called “senile paraplegia” mentioned in the older literature) have received little attention compared to other areas of the body, such as the heart and brain. Spinal cord infarctions are thought to account for only 1% of all strokes [19]. Hughes and Brownell postulated that the spinal cord is much less susceptible to atherosclerosis than the brain due to its complex blood supply (Figures 5-7) and a tendency toward developing collateral flow [20]. However, the morbidity and mortality related to spinal cord ischemia may be overlooked due to the difficulty of diagnosis [21]. Also, a condition such as atherosclerosis may not be acutely fatal but is capable of leading to a myriad of other conditions that are more likely to be diagnosed at post-mortem autopsy, such as thrombosis, embolus, aneurysm, or anterior spinal artery syndrome (Spiller syndrome) [6, 22-23].

Hughes and Brownell reported five elderly patients with the main presentation abnormality being paresis (four with quadriparesis and one with paraparesis), which could not be accounted for by any other motor neuron or neurological disorders [4]. The weakness was found to be asymmetrical and accompanied by hyperreflexia or areflexia, as well as muscle wasting [4]. At necropsy, cerebral and spinal cord atrophy was visible, and the cerebral arteries displayed signs of atheromatous plaques [4]. Microscopy revealed focal necrosis of both the gray and the white matter, degenerative softening of white matter, Wallerian degeneration of long tracts, and gliosis and neuronal death. These findings were accompanied with severe, systemic atherosclerosis and hyaline thickening and mural changes of the capillaries and small vessels within the gray and white matter [4]. These findings prompted the authors to hypothesize the existence of a neurologic syndrome caused by spinal cord ischemia from atherosclerosis [4]. The only findings seen in our cadaveric spinal cord specimens were due to normal aging or were nutritionally related (e.g., vitamin B12 deficiency). No findings consistent with ischemia were found. Additionally, none of our specimens had atherosclerosis of the radicular or spinal arteries.

Another such case study report by Fieschi, et al. attempted to look at ischemic lesions of the spinal cord and the degree of atherosclerosis present in undamaged segments of the spinal cord from 10 elderly subjects who died of acute cerebrovascular pathology [5]. A single lesion was found in four of the subjects, and another subject was found to have two lesions [5]. Of the six lesions, two were found at the level of C8, two were found at the level of T5, one at the level of L2, and one at the conus medullaris [5]. Three of the lesions were located at the base of the anterior and lateral horns, one was at the base of the posterior horn, and one in the center of the anterior horn [5]. All lesions showed gross rarefaction, necrosis of neurons, and weakened staining [5]. Nine of the subjects were found to have gross atherosclerosis of the aorta and other extraspinal vessels [5]. Six cases revealed sclerotic changes of the radicular arteries, but only three cases showed any mural thickening of the anterior spinal artery [5]. Most of the lesions were located within the intramedullary network, but no arteries were found to be completely occluded [5]. The authors concluded that their findings indirectly supported the theory of vascular myelopathy due to ischemia of the spinal cord in the elderly [5].

Jellinger, et al. found atherosclerotic changes of major spinal vessels in 12.7% of their specimens and 27.1% of subjects older than 61 years old [6]. Unlike findings from previous case studies, atherosclerotic plaques were rarely seen in the intramedullary network and radicular arteries, yet diffuse fibrosis was found throughout the spinal arteries at all levels of the spinal arterial network [6]. However, this fibrosis was rarely associated with vessel stenosis and was correlated with advancing age [6]. There was also a significant correlation between fibrosis of the small intramedullary vessels and myelopathy, but the significance of the correlation decreased from the cervical region to the conus medullaris [6]. Within the spinal arterial system, the anterior spinal artery was affected most frequently [6]. From these data, the authors hypothesized that the smaller lumen of the intramedullary vessels produced less space for turbulent flow to occur, thus reducing shear stress and preventing atherosclerotic plaque formation [6]. As in the previously presented case studies, the common lesions found in the spinal cord consisted of focal necrosis with rarefaction [6]. Jellinger, et al. concluded that while spinal cord atherosclerosis may not be as prevalent as other areas of the cardiovascular system, it is still far more common than previously thought and was noted in well over a quarter of the total population sampled [6]. Atherosclerosis of the aorta was found to be more frequent than in the spinal arteries and displayed the greatest correlation with the observed myelopathy [6]. This led the authors to theorize about the impact of atherosclerosis of the larger extraspinal vessels on spinal cord degeneration [6]. However, no correlation was established between systemic atherosclerosis and the fibrosis found within the spinal vessels [6]. Likewise, Turnbull, et al. found no evidence of arterial narrowing due to atherosclerosis in the capillary or central arterial network of the spinal cord [10]. The authors suggested that the numerous extraspinal vessels and hemodynamics protected these vessels from the effects of atherosclerosis. The findings of Jellinger, et al. are contrasted by a more recent study by Wang, et al., in which the pathological changes in spinal cords of 19 centenarians were studied [24]. While Wang, et al. found microscopic evidence of atherosclerosis in 13 subjects, they found no neuronal degradation due to vascular disturbances [24]. The study did identify areas of myelin loss, neuronal damage, and Wallerian degeneration, but the majority of these proved to be a result of degeneration/disorders of the spinal canal [24]. Although some degree of atherosclerosis of the aorta was seen among all specimens, we did not find any spinal cord artery that had obvious atherosclerosis.

Systemic and spinal cord atherosclerosis play a role in the development of anterior spinal artery syndrome [15, 22-23, 25-26]. In addition to atherosclerosis, anterior spinal artery syndrome can be caused by syphilis, trauma, cross-clamping of the aorta during surgery, intervertebral disc disease, osteophytes, spondylosis, aneurysm, compression by neoplasm, or congenital malformation [15, 22-23, 25-26]. It commonly presents with acute neck pain, paresthesia, flaccid paralysis that may progress to spastic paralysis, loss of bowel and bladder control, loss of temperature sensation, and loss of pain sensation [15, 22-23, 25-26]. These symptoms are accompanied by glial scarring and necrosis of the gray matter supplied by the anterior spinal artery [15, 22-23, 25-26]. Interestingly, none of our specimens was found to have atherosclerotic involvement of the anterior spinal artery or its branches.

Atherosclerosis has always been one of the more common etiologies of anterior spinal artery syndrome, especially with insidious onset, but early case studies and research illustrated the impact of systemic atherosclerosis on hypoperfusion of the spinal cord [15, 22-23, 25-26].  Skinhoj presented three cases of pure anterior spinal artery syndrome with an insidious onset that could not be explained by another etiology [15]. Upon further investigation, the patients were found to have extensive aortic calcification from atherosclerosis [15]. Similar cases were published by O’Moore and Laguna and Cravioto, who demonstrated aortic calcification in patients with unexplainable anterior spinal artery syndrome [25-26]. More recent studies by Nedeltchev, et al. and de la Barrera, et al. also confirmed that atherosclerosis and its sequelae are the most common cause of anterior spinal artery syndrome and cause over one-third of all cases [22-23].

Our cadaveric results do not support these conclusions because no signs of ischemia were found in any specimen. Other factors, including vessel compliance, blood pressure, and vascular anastomoses (e.g., collateral circulation), which were not analyzed in our study, are probably involved in maintaining cord perfusion in the face of narrowed parent and spinal arteries.

## Conclusions

Many theories have been suggested as to why the spinal arterial network itself is only minimally affected by atherosclerosis. Reason would suggest that, due to the vast number of bifurcations in the spinal arterial system, it would be plagued by diffuse plaque formation and arterial narrowing caused by the turbulent flow of blood and the resultant shear stress, which is seen in other areas of the circulatory system. However, this is not the case. Some researchers theorize that the minute luminal diameter limits turbulent flow and that a lower pressure environment is not as conducive to the genesis of large plaque formation. Still other theories suggest that the expansive nature of the spinal cord vasculature provides more than ample collateral circulation despite any stenosis that may exist. The watershed zones between intramedullary vessels allow for a protective “double-coverage” that ensures adequate profusion of tissues. Our findings demonstrate that plaque disease in adjacent arteries is not predictive of signs of spinal cord ischemia.
